# Intermittent face cooling reduces perceived exertion during exercise in a hot environment

**DOI:** 10.1186/s40101-021-00262-0

**Published:** 2021-09-06

**Authors:** Taiki Miyazawa, Mirai Mizutani, John Patrick Sheahan, Daisuke Ichikawa

**Affiliations:** 1 Department of Health and Sports Science, Faculty of Wellness, Shigakkan University, Obu, Japan; 2 Department of Physical Education, Shigakkan University Junior College, Obu, Japan; 3 Institute of Sport Science, Yamanashi Gakuin University, Kofu, Japan; 4 Department of Biomedical Engineering, Toyo University, Kawagoe, Japan

**Keywords:** RPE, Temperature regulation, Intermittent cooling, Endurance exercise, Performance

## Abstract

**Background:**

Facial cooling (FC) is effective in improving endurance exercise performance in hot environments. In this study, we evaluated the impact of intermittent short-lasting FC on the ratings of perceived exertion (RPE) during exercise.

**Methods:**

Ten healthy men performed 40 continuous minutes of ergometric cycle exercise at 65% of the peak heart rate in a climatic chamber controlled at an ambient temperature of 35 °C and a relative humidity of 50%. In the control (CONT) trial, the participants performed the exercise without FC. In two cooling trials, each participant underwent 10 s of FC at 2- (FC2) and 4-min (FC4) intervals while continuing to exercise. FC was achieved by applying two soft-gel packs (cooled to 0 °C) directly and bilaterally on the forehead, eyes, and cheeks. In another cooling trial, 10 s of FC was performed at 2-min intervals using two soft-gel packs cooled to 20 °C (FC2-20).

**Results:**

The RPE values in the FC4 trial were significantly lower than those in the CONT trial at 20 min (FC4, 11.6 ± 2.2 points; CONT, 14.2 ± 1.3 points; *P* < 0.01). Further, significant differences in the RPE values were observed between the FC4 and CONT trials at 5–15 min and 25–40 min (*P* < 0.05). RPE values were also significantly lower in the FC2 trial than in the CONT trial (5–40 min). Although the RPE values in the FC2-20 trial were significantly lower (5–10 min; 15–20 min) than those in the CONT trial, there were no significant differences in the RPE between the FC2-20 and CONT trials at 25–40 min. At 35 min, the RPE values were significantly higher in the FC2-20 trial than in the FC2 trial (*P* < 0.05).

**Conclusion:**

Intermittent short-lasting FC was associated with a decrease in RPE, with shorter intervals and lower temperatures eliciting greater attenuation of increase in the RPE.

## Background

In hot environments, physical exercise can lead to significant increases in core temperature and hypohydration. These effects can, in turn, lead to hyperthermia and dehydration, which can eventually impair and limit endurance performance [1–6]. Such decreases in performance may be induced by physiological changes such as cardiovascular strain [7–9], glycogen depletion [10], central nervous system dysfunction [11], and perceptual discomfort [12–15]. The Summer Olympic Games and the World Athletics Championships are held during the summer, typically from July to August. Changing the schedules of major sports events due to weather concerns is virtually impossible as the dates are decided several years in advance. Hence, it is essential to effectively manage the risk of heat-related concerns in exercising athletes.

Researchers have investigated a variety of cooling methods for reducing health risks and optimizing exercise performance, which differ in duration, method, and site of application [16]. Compared to other areas of the body, the facial region tends to be more sensitive to cooling due to its greater density of cold-sensitive afferent thermal receptors and its cutaneous sudomotor/alliesthesial thermosensitivity to cooling [17–19]. Previous studies have reported that selective facial cooling (FC) is particularly effective in improving endurance exercise performance in hot environments, increasing exercise time to fatigue [20] and lowering ratings of perceived exertion (RPE) [20, 21]. Such improvements in performance may be related to FC-induced beneficial changes in central fatigue [22], cerebral blood flow [23, 24], and brain temperature [25]. However, previous studies concerning the effects of FC on endurance performance have failed to consider real-world sports situations. In these studies, FC was continuous during the exercise period.

Given that it is difficult to cool the facial region continuously during athletic competitions and training, short-lasting FC, which can potentially be implemented during exercise, may be more applicable to real-world situations. A previous study has reported that intermittent facial water spraying (22 °C) during a 5-km running time trial in the heat (33 °C) resulted in improved performance, compared to that with no cooling [26]; however, the effects of intermittent short-lasting FC during exercise have yet to be elucidated completely. Depending on the conditions, conducting intermittent short-lasting FC at frequent intervals and controlling cooling temperatures in real-world situations is considered difficult. Therefore, it is necessary to identify the length of intervals and the optimal temperature for short-lasting FC that could bring about a positive effect.

In this study, we aimed to determine the length of short-lasting FC intervals and FC temperatures that would be effective in reducing physiological and/or psychological stress during exercise in the heat, based on the assumption that such reductions could potentially improve endurance performance. We hypothesized that intermittent FC could attenuate increases in RPE during exercise, and that shorter intervals between short-lasting FC would elicit more intense effects on the RPE. Furthermore, we examined whether there were variations in the effectiveness of intermittent FC at different temperatures.

## Methods

### Participants and ethical approval

Ten healthy men with a mean (±standard deviation [SD]) age of 21 ± 1 years, height of 171 ± 6 cm, body mass of 68 ± 5 kg, and body mass index of 23 ± 2 kg/m^2^ voluntarily participated in this study. Each participant provided written informed consent after all potential risks and procedures were explained. All experimental procedures and protocols conformed to the Declaration of Helsinki and were approved by the Institutional Review Board of the Faculty of Wellness at Shigakkan University (IRB # 99). None of the participants were taking any medication that may have influenced their hemodynamic responses to exercise. All participants were familiarized with the equipment and procedures prior to any experimental sessions. Peak heart rate (HR_peak_) was determined using an incremental protocol on a cycle ergometer (Corival cpet; Lode, Netherlands) 1 week before the main experiments. Participants were exposed to an initial work rate of 30 W at a pedal rate of 60 revolutions∙min^−1^. They were instructed to maintain the frequency of pedaling, and the work rate was increased by 15–20 W every minute until volitional exhaustion. HR was monitored using a two-lead electrocardiogram (ECG). The highest HR value obtained over a 60-s interval was regarded as HR_peak_.

### Experimental design

All experiments were conducted in the morning. The participants finished a light breakfast by 8 a.m. and reported to the laboratory at 10 a.m. They were requested to avoid caffeinated beverages, alcohol, and strenuous physical activity for at least 24 h before the experiment. Following the application of the instrumentation, participants rested quietly in a seated position without a backrest, allowing for cardiovascular stability. After 5-min baseline (BL) data collection, the participants performed 40 min of ergometric cycle exercise in the sitting position at 65% HR_peak_ with a pedaling frequency of 60 revolutions∙min^−1^. The exercise duration and intensity were set based on the reference of the previous study that investigated the effects of continuous FC [21]. The mean (± SD) intensity was 144 ± 4 W.

In the control trial (CONT), the participants completed the exercise session without FC. In two cooling trials, each participant underwent 10 s of FC at intervals of 2 (FC2) and 4 min (FC4) while continuing to exercise. FC was achieved using two soft-gel packs (15 × 20 cm) cooled to 0 °C and applied directly and bilaterally on the forehead, eyes, and cheeks, as these areas exhibit the highest sensitivity to cooling [27, 28]. In another cooling trial, each participant underwent 10 s of FC at 2-min intervals with two soft-gel packs cooled to 20 °C (FC2-20) and applied during exercise. The FC intervals were determined based on the effective playing duration in soccer games. Effective playing duration indicates the duration of play after subtracting the amount of time in which the ball is out of play. In a 90-min soccer game, the effective playing duration is reportedly ~ 60 min [29]. Thus, application of short-time (< 1 min) cooling procedures using pitch-side water bottles could be conducted every 2 min. The 4-min FC interval was set in consideration of the reduced frequency of cooling procedures. The FC temperatures were determined based on the assumption that iced and room-temperature water would be used for the cooling procedures using pitch-side water bottles.

The participants were instructed to breathe as normally as possible throughout the trials. All trials were carried out in a climatic chamber (TBR-6W2S2L2M; Espec Co., Osaka, Japan) controlled at an ambient temperature of 35 °C and a relative humidity of 50%.

### Measurements

Ear canal temperature (*T*_ear_), skin temperatures, and HR at specific time points in the testing protocol were expressed as the mean of 1 min of data collected at 5-min intervals.

*T*_ear_ was measured using a thermistor in a polyethylene tube (LT-2N-13; Gram Co., Saitama, Japan). The tip of the tube was advanced into the ear canal and sealed from the environment using a cotton patch. Skin temperature was measured using thermistors (ITP082-24; NIKKISO-THERM Co., Tokyo, Japan) placed on the center of the forehead (*T*_face_), the right side of the upper arm (*T*_arm_), chest (*T*_chest_), thigh (*T*_thigh_), and leg (*T*_leg_). During the 10 s of FC, the thermistor measuring the *T*_face_ was covered with the cooled packs. The selected location of the thermistor for measuring *T*_face_ was based on a previous study that investigated the effects of FC during hyperthermic exercise [21]. The mean skin temperature (*T*_sk_) was calculated based on the body surface area distribution and thermal sensitivity of each skin area using the following formula, which was proposed by Ramanathan (1964) [30]:

$$ {T}_{sk}=0.3\times \left({T}_{arm}+{T}_{chest}\right)+0.2\times \left({T}_{thigh}+{T}_{leg}\right) $$

The site of probe placement on the skin was identical for each participant. All of the measurement data were sampled continuously at 1 kHz using an analog-to-digital converter (PowerLab, AD Instruments, Milford, MA, USA) interfaced with a computer.

Body weight loss was estimated using the change in dry body weight measured immediately before and after the experiment.

The RPE data were recorded every 5 min during exercise using a 6-20-point scale [31].

### Statistics

Statistical analysis was performed using SPSS 25 (SPSS, Chicago, IL, USA). After confirming the normality of the distribution using the Shapiro-Wilk *W* tests, the data were analyzed via a two-way (trial-by-time) repeated measures analysis of variance (ANOVA) with a post hoc Tukey’s test. Data are expressed as the mean ± SD, with significance for all two-tailed tests set at *P* < 0.05.

## Results

The actual ambient temperature and relative humidity were 34.9 ± 0.1 °C and 49.9 ± 0.5%, respectively, in the CONT trial, 34.9 ± 0.1 °C and 50.0 ± 0.5 %, respectively, in the FC4 trial, 34.9 ± 0.1 °C and 49.9 ± 0.4 %, respectively, in the FC2 trial, and 35.0 ± 0.1 °C and 49.9 ± 0.4%, respectively, in the FC2-20 trial. There were no significant differences in the ambient temperature and relative humidity among the trials.

**Fig. 1:**
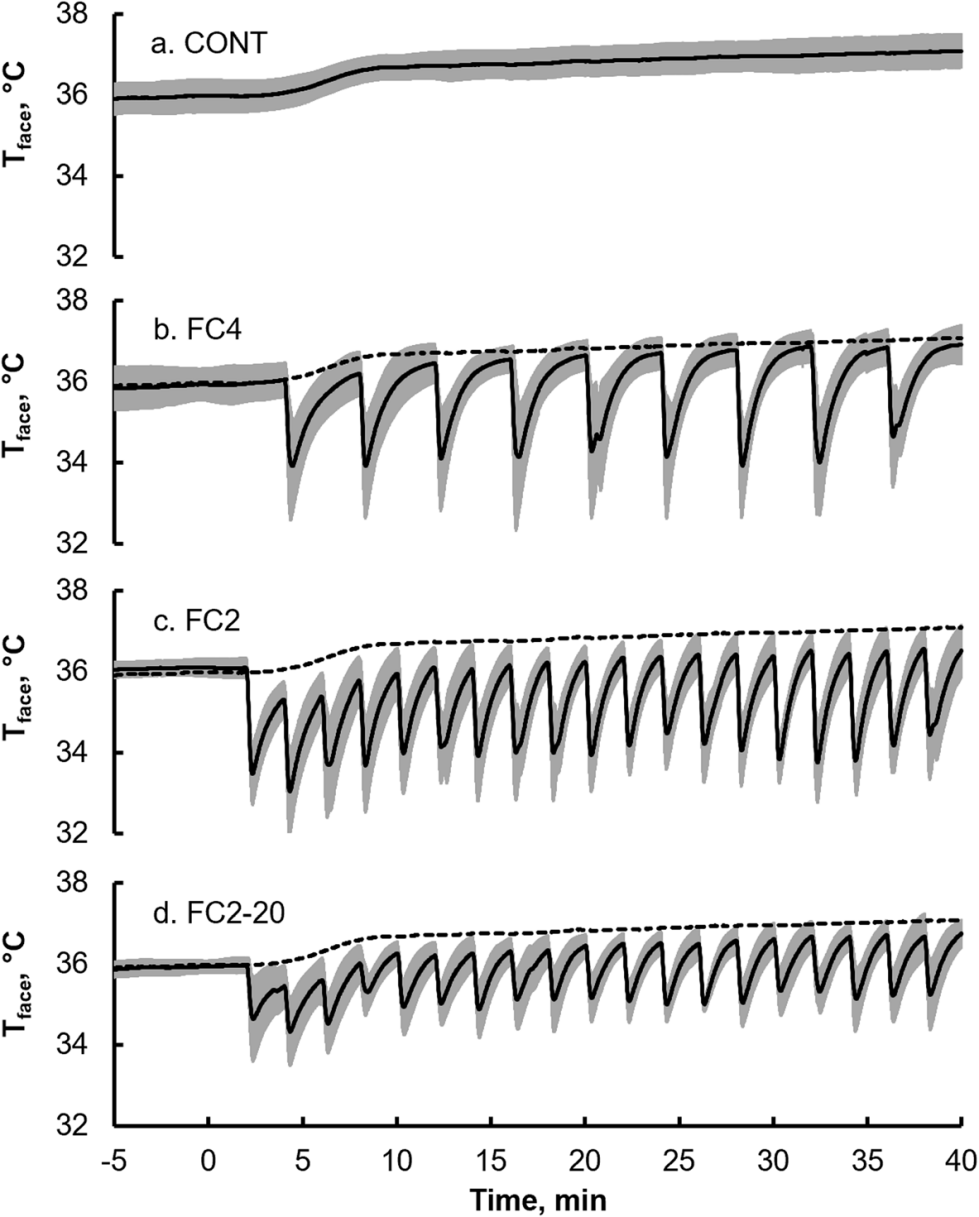
Forehead temperature (*T*_face_) in the CONT (**a**), FC4 (**b**), FC2 (**c**), and FC2-20 trials (**d**). Dashed lines in **b**, **c**, and **d** represent the CONT trial data. Values represent the mean ± SD (gray zone) for 10 participants. CONT, control exercise trial; FC4: facial cooling with 0 °C packs at 4-min intervals; FC2: facial cooling with 0 °C packs at 2-min intervals; FC2-20: facial cooling with 20°C packs at 2-min intervals

The time course of *T*_face_ is shown in Fig. 1. In the CONT trial, we observed continuous increase in the T_face_ during exercise. In response to intermittent short-lasting FC during exercise, the *T*_face_ rapidly decreased in the FC4, FC2, and FC2-20 trials.

Changes in the *T*_face_ with FC are shown in Table 1. The *T*_face_ before FC in Table 1 represents the average *T*_face_ immediately before all FC during each trial. The *T*_face_ after FC in Table 1 represents the average *T*_face_ 20 s after the beginning of FC, as the lower value of the decrease in the *T*_face_ appeared at around that time. The *T*_face_ decrease with FC in Table 1 represents the difference between *T*_face_ before and after FC. The *T*_face_ before FC in the FC2 trial was significantly lower than that in the CONT trial (36.2 ± 0.4 vs. 36.8 ± 0.4 °C, *P* < 0.05). This implies that the *T*_face_ in the FC2 trial rapidly decreased before reaching approximately the same level as the *T*_face_ in the CONT trial due to subsequent cooling, whereas the *T*_face_ during the FC4 and FC2-20 trials recovered to approximately the same level as the *T*_face_ in the CONT trial. In the FC4, FC2, and FC2-20 trials, the *T*_face_ after FC were significantly (*P* < 0.05) lower than that in the CONT trial. Further, the *T*_face_ after FC in the FC2-20 trial was significantly higher than that in the FC2 trial (35.0 ± 0.5 vs. 33.9 ± 0.6 °C, *P* < 0.05). The *T*_face_ decrease with FC were significantly larger in the FC4, FC2, and FC2-20 trials than that in the CONT trial. In the FC2-20 trial, the *T*_face_ decrease with FC was less than that in the FC2 trial (1.3 ± 0.4 vs. 2.2 ± 0.5 °C, *P* < 0.05).

**Table 1:** Changes in forehead temperature (*T*_face_) with facial cooling (FC)

Variables	CONT	FC4	FC2	FC2-20
*T*_face_ at BL, °C	35.9 ± 0.4	35.9 ± 0.5	36.1 ± 0.2	35.9 ± 0.2
*T*_face_ before FC, °C	36.8 ± 0.4	36.6 ± 0.4	36.2 ± 0.4^#^	36.3 ± 0.3
*T*_face_ after FC, °C	36.7 ± 0.4	34.2 ± 1.1^#^	33.9 ± 0.6^#^	35.0 ± 0.5^#*^
*T*_face_ decrease with FC, °C	0.0 ± 0.0	2.3 ± 1.0^#^	2.2 ± 0.5^#^	1.3 ± 0.4^#*^

Values are means ± SD; *T*_face_ at BL, forehead temperature during base line; *T*_face_ before FC; average forehead temperature immediately before all facial cooling during each trial; *T*_face_ after FC, average forehead temperature 20 s after the beginning of facial cooling; *T*_face_ decrease with FC, difference between forehead temperature before and after facial cooling; CONT, control exercise trail; FC4, facial cooling with 0 °C packs at 4-min intervals; FC2, facial cooling with 0 °C packs at 2-min intervals; FC2-20, facial cooling with 20 °C packs at 2-min intervals. ^#^*P* < 0.05, significant difference compared to the CONT trial. ^*^P < 0.05, significant difference compared to the FC2 trial

**Fig. 2:**
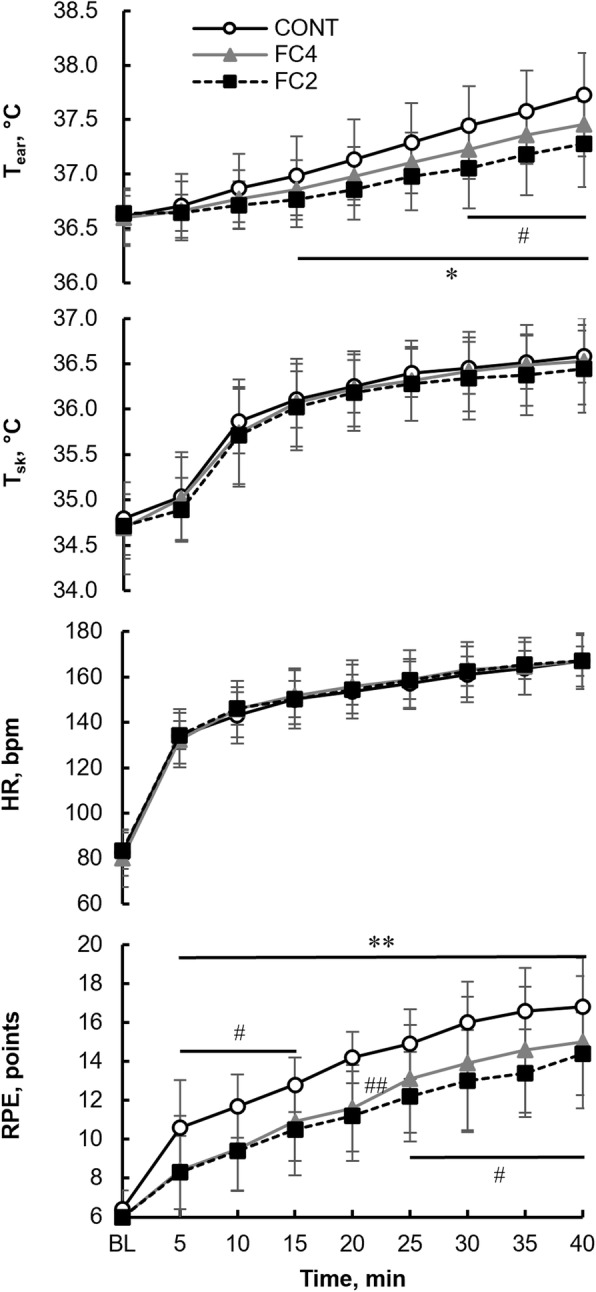
Time course of *T*_ear_, *T*_sk_, HR, and RPE in the CONT (white circle), FC4 (gray triangle), and FC2 trials (black quadrangle). Values are presented as the mean ± SD for 10 participants. ^#^*P* < 0.05 and ^##^*P* < 0.01, significant difference between the CONT and FC4 trials. *P < 0.05 and **P < 0.01, significant difference between the CONT and FC2 trials. *T*_ear:_ ear temperature; *T*_sk_: skin temperature, HR: heart rate; RPE: ratings of perceived exertion; CONT, control exercise trial; FC4: facial cooling with 0 °C packs at 4-min intervals; FC2: facial cooling with 0 °C packs at 2-min intervals

We then compared the effect of interval length between the short-lasting FC and the CONT trial. Figure 2 illustrates the time course of the *T*_ear_, *T*_sk_, HR, and RPE in the CONT, FC4, and FC2 trials.

The T_ear_ was significantly increased (*P* < 0.01) after 15 min of exercise (37.0 ± 0.4 °C), compared with the BL values (36.6 ± 0.3 °C) in the CONT trial and reached 37.7 ± 0.4 °C at 40 min. In the FC4 and FC2 trials, significant increases in the *T*_ear_ were observed at 25 min and 20 min, respectively. The T_ear_ was also significantly lower in the FC4 and FC2 trials than in the CONT trial (*P* < 0.05) from 30–40 min and 15–40 min, respectively. At 40 min, the *T*_ear_ in the FC4 and FC2 trials reached 37.5 ± 0.3 °C and 37.3 ± 0.4 °C, respectively.

The *T*_sk_ and HR increased significantly (*P* < 0.01) from the BL values, beginning at 10 min and 5 min in all trials, respectively. There were no significant differences in the *T*_sk_ and HR among the trials at each time point.

After 5 min, the RPE had significantly increased compared with the BL values (6.4 ± 1.0 points) in the CONT trial (10.6 ± 2.5 points, *P* < 0.01). Although the RPE in the FC4 and FC2 trials also increased with exercise, significant differences (*P* < 0.01) from the BL values were observed in both trials after 10 min (FC4, 9.5 ± 2.1 points; FC2, 9.4 ± 2.1 points). During exercise, the RPE values were significantly lower in the FC4 trial (5–15 min and 25–40 min, *P* < 0.05; 20 min, *P* < 0.01) than those in the CONT trial, reaching 15.0 ± 3.4 points at 40 min. The RPE values were also significantly lower in the FC2 trial than in the CONT trial (5–40 min, *P* < 0.01). The mean RPE value in the FC2 trial at 40 min was 14.4 ± 2.1 points.

There were no significant differences between the FC4 and FC2 trials in the *T*_ear_, *T*_sk_, HR, or RPE values at the same time points.

**Fig. 3:**
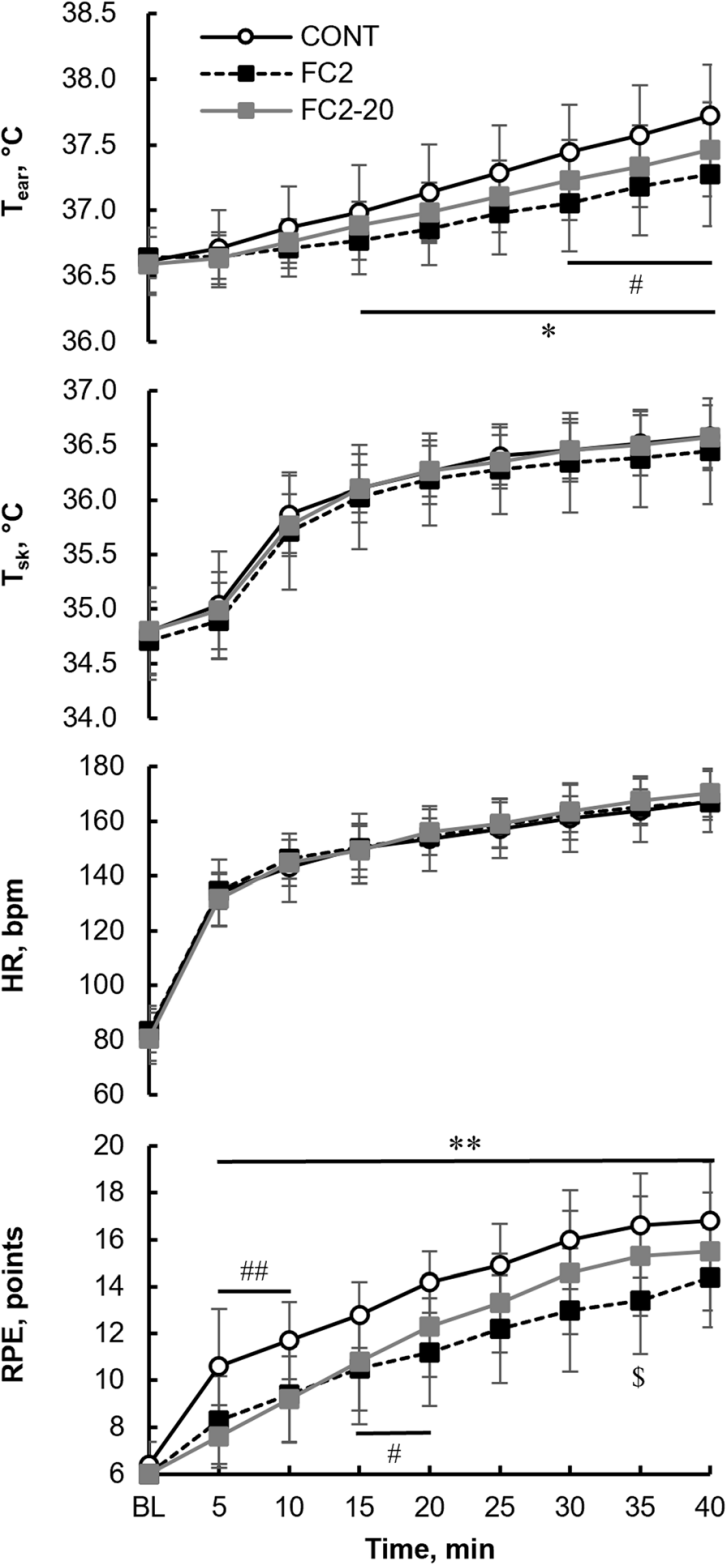
Time course of *T*_ear_, *T*_sk_, HR, and RPE in the CONT (white circle), FC2 (black quadrangle), and FC2-20 trials (gray quadrangle). Values are presented as the mean ± SD for 10 participants. ^#^*P* < 0.05 and ^##^*P* < 0.01, significant difference between the CONT and FC2-20 trials. *P < 0.05 and **P < 0.01, significant difference between the CONT and FC2 trials. ^$^*P* < 0.05, significant difference between the FC2 and FC2-20 trials. *T*_ear:_ ear temperature; *T*_sk_: skin temperature, HR: heart rate; RPE: ratings of perceived exertion; CONT, control exercise condition; FC4: facial cooling with 0°C packs at 4-min intervals; FC2: facial cooling with 0 °C packs at 2-min intervals

We also examined the effects of different cooling temperatures on the RPE. Figure 3 illustrates the time course of the *T*_ear_, *T*_sk_, HR, and RPE in the CONT, FC2, and FC2-20 trials. The values for the CONT and FC2 trials were the same as those shown in Fig. 2.

In the FC2-20 trial, the *T*_ear_ values were significantly increased (*P* < 0.01) compared with the BL values (36.6 ± 0.2 °C) at 15 min (36.9 ± 0.2 °C) and were significantly lower (*P* < 0.05) than those in the CONT trial at 30–40 min. There were no significant differences in the *T*_ear_ values between the FC2-20 and FC2 trials at the same time points.

In addition, the T_sk_ and HR values in the FC2-20 trial were significantly increased at 10 min and 5 min, respectively (*P* < 0.01), compared with the BL values. There were no significant differences in the *T*_sk_ or HR among the trials at each time point.

The RPE increased with exercise during the FC2-20 trial, and significant differences were observed at 10 min (9.2 ± 1.8 points) (*P* < 0.01), compared with the BL (6.0 ± 0.0 points) values. Although the RPE values in the FC2-20 trial were significantly lower (5–10 min, *P* < 0.01; 15–20 min, *P* < 0.05) than those in the CONT trial, there were no significant differences in the RPE between both trials at 25–40 min. At 35 min, the RPE was significantly higher (*P* < 0.05) in the FC2-20 trial (15.3 ± 2.5 points) than that in the FC2 trial (13.4 ± 2.3 points).

There were no significant differences between the FC2 and FC2-20 trials in the *T*_ear_, *T*_sk_, or HR at the same time points.

**Fig. 4:**
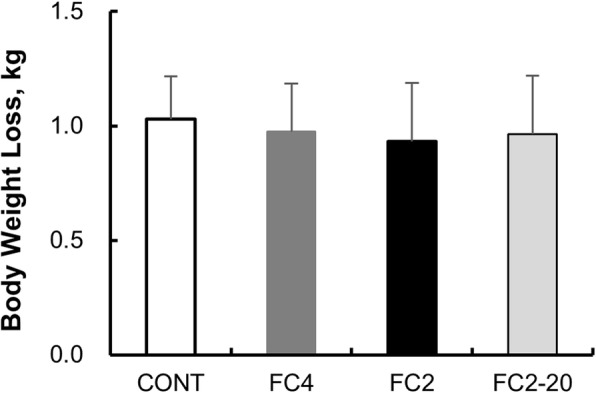
Body weight loss during exercise in the CONT (white), FC4 (gray), FC2 (black), and FC2-20 trials (light gray). Values are presented as the mean for 10 participants. CONT, control exercise trial; FC4: facial cooling with 0°C packs at 4-min intervals; FC2: facial cooling with 0 °C packs at 2-min intervals

No significant differences in body weight loss were observed among the trials (Table 2 and Fig. 4).

**Table 2:** Body weight loss

Variables	CONT	FC4	FC2	FC2-20
BW before experiment, kg	68.0 ± 4.7	68.5 ± 5.4	68.6 ± 5.1	68.6 ± 5.1
BW after experiment, kg	66.9 ± 4.7	67.5 ± 5.5	67.6 ± 5.2	67.6 ± 5.1
Body weight loss, kg	1.03 ± 0.19	0.98 ± 0.21	0.93 ± 0.26	0.97 ± 0.23

Values are means ± SD, *BW*, body weight, *CONT* control exercise trial, *FC4* facial cooling with 0 °C packs at 4-min intervals, *FC2* facial cooling with 0 °C packs at 2-min intervals, *FC2-20* facial cooling with 20 °C packs at 2-min intervals

## Discussion

In this study, we aimed to examine whether intermittent short-lasting FC can impact the RPE during dynamic exercise. As expected, intermittent short-lasting FC attenuated increase in the RPE during exercise in a hot environment. Further, shorter FC intervals and lower cooling temperatures tended to elicit a greater restriction on RPE increases. These findings indicate that intermittent short-lasting FC may be effective in improving exercise performance in hot environments.

Previous studies have demonstrated that cooling the facial region using ice packs or by pouring water on the face may improve endurance exercise performance [20, 21, 26]. In one such study [20], the RPE was significantly lower among participants who were subjected to facial fanning throughout cycling exercises at 75% of maximal oxygen uptake (ambient temperature 29 ± 1.0 °C; relative humidity ~ 50%). Although the detailed numerical values pertaining to the RPE were not shown in the previous study, an RPE figure from the previous study demonstrated that facial fanning elicited a 1–2 points decrease in the RPE compared to that of the control trial without facial fanning; this was in addition to improving the time to exhaustion by 51% [20]. Another previous study has reported that the RPE was significantly reduced at the end of a 40-min exercise period (exercise intensity, 65% of aerobic peak power; ambient temperature, 33 °C; relative humidity, 27%) through FC by spraying a mist of cold water, compared to a trial without FC (14.1 ± 0.8 vs. 15.5 ± 0.7 points) [21]. However, these previous studies adopted continuous FC during exercise, which is not possible during games, competitions, and training in real sports situations. In this study, 10 s of FC at 4- and 2-min intervals (FC4, FC2, and FC2-20) during submaximal cycling exercise in a hot environment significantly attenuated an increase in the RPE. In the FC2 trial, in particular, the RPE at the end of the 40-min exercise period was restricted at 14.4 ± 2.1 points, whereas the RPE in the CONT trial reached 16.8 ± 2.5 points. The magnitudes of change in the RPE between the trials with and without FC were higher in this study than that in the previous study [21]; although notably, the experimental conditions differed slightly between the trials. These results suggest that intermittent short-lasting FC induces improvements in RPE similar to those observed during continuous FC. Thus, intermittent short-lasting FC during exercise may help to improve both athletic performance and efficiency in physical working environments.

This study also investigated the differences in the effects of FC at 4- (FC4) and 2-min (FC2) intervals. Although there were no significant differences in RPE between the FC4 and FC2 trials (Fig. 2), the mean RPE value in the FC2 trial was lower than that in the FC4 trial after 5 min. Furthermore, the magnitude of change in the RPE from the BL value was lower in the FC2 trial than in the FC4 trial. For instance, the change in RPE between BL and the FC2 trial was 7.4 ± 2.3 points, whereas that between BL and the FC4 trial was 8.6 ± 3.3 points at 35 min; at this time point, the difference in RPE between the FC2 and FC4 trials was the largest. Moreover, *p* values for the RPE comparison between the CONT and FC2 trial were < 0.01 at all time points during the exercise, whereas those in the FC4 trial were < 0.05 and > 0.01, except for the value at 20 min. These results suggest that shorter intervals of intermittent FC can lead to greater attenuation of RPE during exercise. Further studies are required to determine the interval duration at which such improvements disappear.

None of the previous studies regarding the effects of FC on exercise performance in a hot environment have compared the effects of different cooling temperatures. In our study, FC was performed at temperatures of 0 °C (FC2) and 20 °C (FC2-20). Although these cold stimulations induced a decrease in the *T*_face_ in both trials, the magnitude of *T*_face_ decrease with FC in the FC2 trial was significantly larger than that in the FC2-20 trial (2.2 ± 0.5 vs. 1.3 ± 0.4 °C, *P* < 0.05). There were no significant differences in the RPE between the FC2-20 and CONT trials at 25–40 min, whereas the RPE values in the FC2 trial were significantly lower (*P* < 0.01) than those in the CONT trial during the whole exercise period. In addition, the RPE in the FC2-20 trial was significantly higher (*P* < 0.05) than that in the FC2 trial at 35 min. These results suggest that a decrease in RPE induced by intermittent short-lasting FC may be more pronounced at lower cooling temperatures. Although there may be some beneficial effects of FC at 20 °C on the exercise performance, our data indicate that intermittent short-lasting FC should be applied at the lowest temperature possible in real-world sports situations and physical work settings to avoid the deleterious effects of a hot environment.

In this study, FC at 2- and 4-min intervals was determined based on the reference of the effective playing duration in soccer games. A 90-min soccer game reportedly has an effective playing duration of ~ 60 min [29]. Thus, application of short-lasting FC using pitch-side iced water bottles or iced water-saturated towels could be feasibly conducted every 2- or 4-min during the soccer game. Furthermore, in this study, the temperatures of FC were determined based on the assumption that iced and room-temperature water would be used for the cooling procedures using pitch-side water bottles. Therefore, RPE inhibition may be possible with intermittent short-lasting FC during a soccer game. Similarly, it might be possible to perform intermittent short-lasting FC during other sports situations, trainings, and occupational settings that have the time to conduct cooling procedures frequently using pitch-side water bottles or by players carrying small water bottles. However, the present study only assessed the impact of short-lasting FC on psychological and physiological measures. It remains unknown whether the short-lasting FC induces improvements of endurance performance. Additionally, the present study has not given consideration to any effect of convective flow that would occur in a real-life sporting scenario. Convective flow can impact the thermal state of the participant [32] and, as a consequence, may impact the effect of short-lasting FC on RPE and exercise performance. Further studies are necessary to identify any additional effects of intermittent short-lasting FC to confirm whether the findings are generalizable to other situations.

Several studies have noted that continuous FC during endurance exercise in a hot environment can improve RPE and attenuate prolactin (PRL) secretion [20–22]. Increases in PRL secretion during exercise in a hot environment are thought to reflect an intolerable thermal strain and decrease the desire to continue exercising, resulting in central fatigue [33–35]. As suggested in previous studies of continuous FC, intermittent short-lasting FC may decrease central fatigue and attenuate PRL secretion, thereby leading to decreases in RPE. However, the present study is limited in that we did not measure PRL concentrations.

Another key limitation of our study is that we did not measure the core or brain temperatures, which are highly reliable. The *T*_ear_ was measured as the core temperature in this study. One reason for doing this was that we wanted to reduce the physiological and psychological burden on the participant, which would presumably be induced by invasive measurements such as esophageal or rectal temperature. Moreover, a psychological burden on the participant might disrupt the measurement of RPE, which was the focus of our study. Another reason was that previous studies have already demonstrated that continuous FC during dynamic exercise has no effect on the rectal temperature [20–22]. Although the T_ear_ in this study was lower in the FC4, FC2, and FC2-20 trials than in the CONT trial, it remains unclear what these results imply. One possibility is that the decrease in the *T*_ear_ with FC may reflect a reduction in the brain or hypothalamic temperature. In that case, it might be suggested that intermittent short-lasting FC induces a decrease in the RPE through the mechanism of selective cooling of the brain. However, researchers have debated the validity of using tympanic or ear canal temperature as a measure of the brain or hypothalamic temperature [36, 37], with strong evidence arguing that tympanic temperature cannot be used as a consistent index of brain temperature [38]. Another possibility is that the *T*_ear_ measurements might have been tainted by changes in the local skin temperature induced by FC. It has been reported that facial cooling during passive or active heating induces significant deviations in the tympanic temperature [39–41]. Thus, intermittent short-lasting FC may have no effect on brain temperature, and FC-induced improvements in the RPE may be mediated by stimulation of the skin afferents [42]. Further analyses are required to identify the mechanisms underlying these FC-induced changes in RPE.

## Conclusion

In summary, our findings demonstrated that intermittent short-lasting FC may attenuate an increase in the RPE during endurance exercise in a hot environment. Further, shorter intervals and lower temperatures of intermittent FC elicited greater effects on the RPE. These findings suggest that intermittent short-lasting FC during exercise may help in improving psychological stress in real-world sports situations and physical work environments. To apply intermittent short-lasting FC to other sports situations and occupational settings, further analysis is required regarding exercise performance, accurate core body temperature, and convective flow.

## Abbreviations

BL: Baseline
CONT: Control
FC: Facial cooling
HR_max_: Maximum heart rate
PRL: Prolactin
RPE: Ratings of perceived exertion
SD: Standard deviation
*T*_ear_: Ear canal temperature
*T*_arm_: Upper arm temperature
*T*_sk_: Mean skin temperature
WAC: World Athletics Championships
